# Testing for goodness rather than lack of fit of an X–chromosomal SNP to the Hardy-Weinberg model

**DOI:** 10.1371/journal.pone.0212344

**Published:** 2019-02-21

**Authors:** Stefan Wellek, Andreas Ziegler

**Affiliations:** 1 Department of Biostatistics, CIMH Mannheim, Mannheim Medical School of the University of Heidelberg, Mannheim, Germany; 2 Department of Medical Biostatistics, Epidemiology & Informatics, University Medical Center of the Johannes Gutenberg University Mainz, Mainz, Germany; 3 Institute of Medical Biometry and Statistics, University of Lübeck, Lübeck, Germany; 4 StatSol, Moenring 2, Lübeck, Germany; 5 School of Mathematics, Statistics and Computer Science, University of KwaZulu-Natal, Pietermaritzburg, South Africa; Universitat Pompeu Fabra, SPAIN

## Abstract

The problem of checking the genotype distribution obtained for some diallelic marker for compatibility with the Hardy-Weinberg equilibrium (HWE) condition arises also for loci on the X chromosome. The possible genotypes depend on the sex of the individual in this case: for females, the genotype distribution is trinomial, as in the case of an autosomal locus, whereas a binomial proportion is observed for males. Like in genetic association studies with autosomal SNPs, interest is typically in establishing approximate compatibility of the observed genotype frequencies with HWE. This requires to replace traditional methods tailored for detecting lack of fit to the model with an equivalence testing procedure to be derived by treating approximate compatibility with the model as the alternative hypothesis. The test constructed here is based on an upper confidence bound and a simple to interpret combined measure of distance between true and HWE conforming genotype distributions in female and male subjects. A particular focus of the paper is on the derivation of the asymptotic distribution of the test statistic under null alternatives which is not of the usual Gaussian form. A closed sample size formula is also provided and shown to behave satisfactorily in terms of the approximation error.

## Introduction

The Hardy–Weinberg law belongs to the key concepts in genetic epidemiology [1]. Departure from Hardy–Weinberg equilibrium (HWE) can be caused by factors such as inbreeding, assortative mating, selection or migration [2]. The effect of these factors on HWE can be expected to be small in most human populations although selection may play an important role in infectious diseases [2]. Another reason is population stratification which causes a deficit of heterozygotes. Population stratification can be controlled for by methods, such as genomic control (for a detailed overview see e.g., [3]). The presence of copy number variations generally leads to an excess of heterozygotes. Finally, deviation from HWE may be simply caused by genotyping errors. We have previously argued that deviations from HWE should be investigated only in controls for case-control studies and in the entire cohort in cohort studies [2]. For autosomal loci, several “how to guides” have been published for assessing deviation from HWE [4, 5]. These approaches are commonly used as part of the regular quality control in genome-wide association studies and meta-analyses.

For testing deviation from HWE for X-chromosomal markers, no such guidelines are available although testing for HWE is used for quality control on the X-chromosome as well [6]. The complicating factor for assessing deviation from HWE is that males are hemizygous, thus have only one allele on X-chromosomal markers outside of the pseudoautosomal regions, while females have two alleles as on autosomes. Some software packages therefore ignore male subjects and conduct a test for HWE in females only [5]. However, this reduces the sample size and results in a loss of power. Furthermore, an X-chromosomal marker can only be in HWE if the allele frequencies are equal in males and females. If males are neglected, deviation from HWE cannot be thoroughly investigated. Other software packages ignore the difference between autosomal and X-chromosomal markers (see the genetics package in R). As described by [5], these tests are potentially misleading due to coding the genotype of a hemizygous male either as AA or aa as in the standard data format.

The problem of HWE testing on the X chromosome has caught attention quite recently. For example, Graffelman and Weir [7] proposed four frequentist tests for diallelic markers using data from both males and females. An implementation of these procedures is available in an R package called ‘Hardy-Weinberg’ [8]. A Bayesian HWE testing procedure has also been proposed [9]. Other tests are described in the work by Wang et al. [10] and You et al. [11], and an extension to multiallelic markers by Graffelman and Weir [12]. Zheng et al. investigated the impact of deviations from HWE on the properties of association tests for X-chromosomal SNPs [13].

The usual strategy to protect oneself against the distorting effects entailed in violations of the HWE condition consists of filtering markers that do not conform with HWE prior to the conduct of genetic association tests. For autosomal SNPs, i.e., diallelic genetic markers located at an autosome, the traditional statistical procedure to assess HWE is the standard Pearson *χ*^2^-test. Unfortunately, any testing procedure of this type fails to serve the purpose of confirming the compatibility of the marker with the model. Actually, the conventional *χ*^2^-test is tailored for establishing lack rather than goodness of fit, since the statement that the distribution underlying the data is in agreement with HWE plays the role of the null rather than an alternative hypothesis. A significant test result thus indicates incompatibility of the observed data with the model. A well-established way around this basic difficulty inherent in the logic of significance testing is to reformulate and solve the problem of HWE assessment as what is called in biostatistics a problem of equivalence testing (for a systematic treatise on this still fairly fast developing area in statistical methodology, see [14]).

This change of the basic inferential paradigm has been successfully exploited by [15, 16] and [17] for the case of autosomal SNPs. The equivalence tests derived there are tests for goodness rather than lack of fit, in the sense, that they allow one to control the risk of erroneously deciding in favor of the hypothesis that the populations underlying the samples under evaluation are “essentially” compatible with the HWE model. In this phrase, “essentially” means that the deviations between model and truth, if existing at all, are small enough for being treated as marginal and thus irrelevant. Inverting a traditional lack-of-fit test by deciding for the new alternative hypothesis of equivalence between the actual and a HWE-conforming population if it yields a p-value above the conventional significance level of 5%, fails to provide control over the type-I error. In the equivalence setting, the latter consists in incorrectly rejecting the null hypothesis of relevant deviations from the model. The actual size of this risk highly depends on the order of magnitute of the sample size: for small sample sizes, it can become as large as 95%, where for huge sample sizes it approaches zero so that the procedure becomes extremely conservative. The goodness-of-fit test for HWE at autosomal markers constructed by [15] is an exact, uniformly most powerful (UMPU) procedure based on the conditional distribution of the observed number *X*_2_, say, of heterozygotes given the total number *S* of *A*-alleles (with *A* denoting the allele of minor frequency). It rejects if *X*_2_ falls in the interior of some interval whose endpoints depend in a fairly complicated manner on the value of *S* and the significance level (defined as the maximum acceptable probability of incorrectly rejecting the null hypothesis of relevant disequilibrium). In a subsequent paper [17], we were able to show that without substantial loss of power, the exact UMPU test can be replaced with a computationally much simpler approach based on confidence intervals for a function of the population genotype frequencies providing a natural measure of the amount of disequilibrium (the definition of this parametric function will be made precise below in the first subsection of Materials and Methods).

The aim of the present paper is to extend the confidence limit based approach to testing for approximate compatibility of the distribution of some given SNP with the HWE model to the case of X-chromosomal loci. The Materials and Methods (M&M) section, which is the core part of the paper, goes far beyond the description of routine methods of data analysis. It focusses on a rigorous derivation of the newly proposed testing procedure and the formal machinery required for investigating basic properties of the method and planning genetic association studies requiring to ensure the compatibility of sex-linked markers with HWE. It starts with a formally precise description of the equivalence testing approach to HWE assessment for diallelic markers and an extension of the hypothesis formulation to the case that the population under assessment consists of a mixture of allele pairs and single alleles. The proposed way of measuring the amount of disequilibrium jointly for females and males is to define for the two subpopulations separate measures Δ_*f*_ and Δ_*m*_ of the distance of the underlying distribution from the model and to combine these by calculating the ordinary Euclidean distance of (Δ_*f*_, Δ_*m*_) from the origin of the plane. In the Subsection 2 of M&M, we study the asymptotic distribution of the natural estimator of the Euclidean distance of (Δ_*f*_, Δ_*m*_) from **0** obtained by plugging in throughout the observed relative genotype and allele frequencies for the theoretical frequencies (*π*_1_, *π*_2_, *π*_3_) and *p*_*Y*_, respectively. This provides the mathematical basis for the computation of an upper confidence bound to $\Delta ≔\sqrt{\Delta_{f}^{2}+\Delta_{m}^{2}}$, and the corresponding testing procedure, which decides in favor of goodness of fit if this bound falls below the prespecified equivalence margin. In Subsection 3 of M&M, we derive an expression for the exact rejection probability of the goodness-of-fit test under any parameter configuration and establish approximate formulas for the power against different types of alternatives focussing on so-called null alternatives specifying perfect coincidence with the HWE model. In the latter case, which is the most interesting one for applications, the asymptotic distribution of the test statistic is no longer Gaussian and must be established separtely by means of a non-standard construction. The Results section starts with an investigation on level and power of the goodness-of-fit test, which is inherently an asymptotic procedure, in finite samples. Subsequently, the new method is compared to the combined *χ*^2^-test for lack of fit proposed by [7] both for real data taken from a GWAS on venous thrombosis, and simulated data sets. The assessment of the approximate methods of power calculation and the associated sample-size formulas for the new test, is again done by means of exact numerical computation.

## Materials and methods

### Mathematical notation and formulation of the testing problem

The first goodness-of-fit testing procedure made available for purposes of HWE assessment in genetic association studies involving diallelic markers ([15]) was constructed by solving the equivalence problem

$$\begin{array}{c} H:\theta \leq 4/\left(1+\delta_{{}^{\circ}}\right)\;\text{or}\;\theta \geq 4\left(1+\delta_{{}^{\circ}}\right)\;\text{versus}\;K:4/\left(1+\delta_{{}^{\circ}}\right)<\theta <4\left(1+\delta_{{}^{\circ}}\right). \end{array}$$ (1)

In (1), *δ*_°_ stands for a fixed positive constant to be chosen a priori defining the equivalence range for the function

$$\begin{array}{c} \theta =\frac{\pi_{2}^{2}}{\pi_{1}\left(1-\pi_{1}-\pi_{2}\right)}\;\; \end{array}$$ (2)

of the true proportions *π*_1_, *π*_2_, and *π*_3_ of the possible genotypes AA, AB, and BB at the selected locus in the underlying population. The adequacy of the hypotheses formulation (1) for the purpose of establishing goodness rather than lack of fit of an autosomal SNP to HWE is ensured by the following facts:

1. *θ*/4 − 1 has the same sign as $\pi_{2}-2\left(\sqrt{\pi_{1}}-\pi_{1}\right)$, and any genotype distribution with parameter (*π*_1_, *π*_2_, *π*_3_) is in perfect HWE if and only if (*π*_1_, *π*_2_) is a point on the graph of the function
  $$\begin{array}{c} \pi_{2}=2(\sqrt{\pi_{1}}-\pi_{1})\;,\;0<\pi_{1}<1\;. \end{array}$$ (3)
2. For any 0 < *δ*_°_ < 1, there holds the relationship
  $$\begin{array}{c} 4/\left(1+\delta_{{}^{\circ}}\right)<\theta <4\left(1+\delta_{{}^{\circ}}\right)\Leftrightarrow {\tilde{\pi }}_{2}^{l;\delta_{{}^{\circ}}}\left(\pi_{1}\right)<\pi_{2}<{\tilde{\pi }}_{2}^{u;\delta_{{}^{\circ}}}\left(\pi_{1}\right), \end{array}$$ (4)
  where the region bounded by the curves
  $$\begin{array}{c} {\tilde{\pi }}_{2}^{l;\delta_{{}^{\circ}}}\left(\pi_{1}\right)=2(\sqrt{{\left(1+\delta_{{}^{\circ}}\right)}^{-1}\;\pi_{1}\left(1-\delta_{{}^{\circ}}\pi_{1}/\left(1+\delta_{{}^{\circ}}\right)\right)}-\pi_{1}/\left(1+\delta_{{}^{\circ}}\right))\;,\;0<\pi_{1}<1, \end{array}$$ (5)
  $$\begin{array}{c} {\tilde{\pi }}_{2}^{u;\delta_{{}^{\circ}}}\left(\pi_{1}\right)=2(\sqrt{\left(1+\delta_{{}^{\circ}}\right)\;\pi_{1}\left(1+\delta_{{}^{\circ}}\pi_{1}\right)}-\left(1+\delta_{{}^{\circ}}\right)\;\pi_{1})\;,\;0<\pi_{1}<1, \end{array}$$ (6)
  encloses the HWE curve (2).

The family $\left\{M\left(\pi_{1},\pi_{2},\pi_{3}\right)|0<\pi_{1},\pi_{2},\pi_{3}<1,\;\pi_{1}+\pi_{2}+\pi_{3}=1\right\}$ of all trinomial distributions is readily seen to be an exponential family with parameters *θ* (as defined in Eq (2)) and *ϑ* = *π*_1_/*π*_3_. This fact is the starting point for the construction of the optimal—precisely: uniformly most powerful unbiased—solution carried out by [15]. The practical implementation of the UMPU test requires acquaintance with advanced statistical software (in R, the programs gofhwex and gofhwex_1s of the package EQUIVNONINF [18] can be used). Since this might restrict the suitability of the method for routine use in the analysis of large-scale genetic association studies, in a more recent paper [17], we developed an asymptotic testing procedure for the same problem as a more user-friendly alternative. The latter relies on the principle of confidence interval inclusion, which was introduced by [19] into the field of bio-equivalence assessment and can easily be shown (cf. [14], § 7.1) to be a special case of the intersection-union principle (IUP) as formulated by [20]. Despite its conceptual and computational simplicity—a pocket calculator suffices, the IUP-based asymptotic test for (1) turns out to produce rejection regions which, for the sample sizes commonly availabe for genetic association studies, do not differ by more than a practically negligible amount from the critical region of the exact UMPU test for the same setting and specifications.

**Fig 1:**
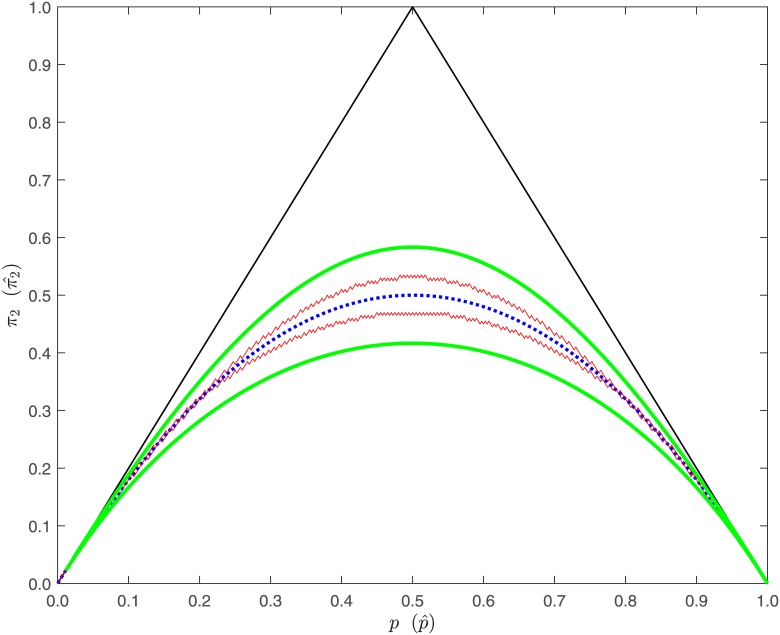
De Finetti diagram of the boundary curves of the equivalence region specified under the alternative hypotheses of (1). Ragged lines: critical region of the test to be generalized for X-chromosomal SNPs. [*δ*_°_ = 0.96, *α* = 0.05, *n* = 200].

Fig 1 illustrates the confidence interval inclusion rule for the case that a sample of size *n* = 200 is available from a genotype distribution of an autosomal SNP and the choice *δ*_°_ = 0.96 for the constant determining the equivalence bounds to *θ* considered acceptable for a SNP in sufficiently good agreement with HWE. Using de Finetti’s coordinate transformation (*π*_1_, *π*_2_)↦(*π*_1_ + *π*_2_/2, *π*_2_), the graph shows the rejection region of the test obtained by checking an asymptotic 95%-confidence interval for *θ* for inclusion in the equivalence interval specified under the alternative hypothesis *K* of (1). As shown by [17], the choice *δ*_°_ = 0.96 can be justified by the fact that the corresponding equivalence margin is the smallest one for which the sample size required to attain a power 90% against the null alternative of perfect agreement with HWE in a test at nominal level *α* = 0.05 does not exceed 3,000, provided a SNP with minor allele frequency satisfying 0.1 ≤ *MA* ≤ 0.5 has to be evaluated.

If A and B are the two possible alleles for a SNP at an X-locus, generalizing the alternative hypothesis of (1) in a natural way leads to replacing *K* by the statement that the values taken in the underlying subpopulations, i.e., female and male subjects, by the following two distance measures are both “sufficiently small”:

Δ_*f*_ = distance among females between the parameter *θ* of (2) or some suitable transform of it, and its value under perfect HWE

Δ_*m*_ = distance between the true distribution of A-alleles among males being binomial with parameter *p*_*Y*_, say, and a binomial distribution having the allele frequency holding for females under perfect HWE as its parameter.

Regarding the female subpopulation, we adopt the confidence-limit based approach to constructing a goodness-of-fit test for HWE assessment with autosomal SNPs in a 1:1 manner. Conceptually, this version of a test for equivalence of the genotype distribution of a diallelic marker under analysis with HWE relies on the following fact: a measure of distance from the model which combines straightforward biological interpretability with mathematical convenience can be based on the difference between half the square root of the parameter *θ* as defined above in Eq (2), and unity. Actually, 1 is the value of $\omega :=\left(1/2\right)\sqrt{\theta }$ in a population being in perfect HWE. With a view to symmetry of the distribution of its natural point estimator, we prefer to replace the parameter *ω*, which we proposed to term relative excess heterozygosity (REH), by its logarithm and to measure in the subpopulation of females the degree of disequilibrium in terms of the distance of log *ω* from zero, i.e., |log *ω*|. Accordingly, we define the first component of an aggregate measure of disequilibrium combining the characteristics of the genotype distributions for both gender strata to be given by

$$\begin{array}{c} \Delta_{f}=|\text{log}\left(\pi_{2}\right)-\left(\text{log}\left(\pi_{1}\right)+\text{log}\left(\pi_{3}\right)\right)/2-\text{log}\left(2\right)|, \end{array}$$ (7)

assuming throughout that the *π*_*j*_ denote the genotype frequencies in the subpopulation of females (with the subscript *f* being omitted for brevity).

As explained above, the other component, Δ_*m*_, must be defined as a function of (*π*_1_, *π*_2_, *π*_3_) and *p*_*Y*_, the true frequency of the allele A of interest in the male subpopulation. In order to make this definition suitable for the present purpose, Δ_*m*_ has to be a reasonable measure of distance between two binomial distributions with parameters *p*_1_ = *π*_1_ + *π*_2_/2 and *p*_2_ = *p*_*Y*_. The literature on equivalence testing methods for clinical trials contains several different proposals for choosing such a measure. As has been argued by [14] (see also [21]), a particularly well suited definition is based on the log odds ratio between *p*_1_ and *p*_2_, which in the present context leads to setting

$$\begin{array}{c} \Delta_{m}=|\text{log}\left(\pi_{1}+\pi_{2}/2\right)-\text{log}\left(1-\pi_{1}-\pi_{2}/2\right)-\text{log}\left(p_{Y}\right)+\text{log}\left(1-p_{Y}\right)|. \end{array}$$ (8)

Thus, as an aggregate criterion of approximate compatibility of an X-chromosomal SNP with HWE to be satisfied under the alternative hypothesis of the test to be subsequently derived, we use the condition

$$\begin{array}{c} \begin{array}{c} {}[{\left(\text{log}\left(\pi_{2}\right)-\left(\text{log}\left(\pi_{1}\right)+\text{log}\left(\pi_{3}\right)\right)/2-\text{log}\left(2\right)\right)}^{2}+(\text{log}(\pi_{1}+\;\\ \pi_{2}/2)-\text{log}\left(1-\pi_{1}-\pi_{2}/2\right)-\text{log}\left(p_{Y}\right)+\text{log}\left(1-p_{Y}\right){)}^{2}{]}^{1/2}<\varepsilon . \end{array} \end{array}$$ (9)

Denoting the signed version of Δ_*f*_ and Δ_*m*_ by $\Delta_{f}^{\pm }$ and $\Delta_{m}^{\pm }$, respectively, the set of all combinations of subpopulation genotype and allele frequencies satisfying (9) obviously corresponds to a circular disc of radius *ε* in the parameter space of $\left(\Delta_{f}^{\pm },\Delta_{m}^{\pm }\right)$ centered at the origin. Hence, it seems reasonable to choose *ε* to be the radius of the smallest circle which contains a square with edges of length being equal to twice a suitable common margin to Δ_*f*_ and Δ_*m*_. In testing for equivalence of two binomial distributions with respect to the log odds ratio, a well-established specification of the equivalence margin is *ε*_*LOR*_ = log(12/8) ≈ 0.41 (for the rationale behind this recommendation (cf. [14], § 1.6). Furthermore, the margin which has been proposed by [17] for *REH* = *ω*^2^ = *θ*/4 is 1.96 corresponding to log(1.4) ≈ 0.34 for Δ_*f*_. Since these margins are not identical we propose to take the tighter one as a basis for specifying the margin *ε* to $\Delta =\sqrt{\Delta_{f}^{2}+\Delta_{f}^{2}}$ so that we propose to set $\varepsilon =\sqrt{2}\text{log}\left(1.4\right)\approx 0.48$.

### Interval estimation and testing procedure

As a pivotal quantity for inference about our distance measure Δ we consider the plug-in point estimator obtained through replacing the population frequencies involved by the homologous empirical proportions

$$\begin{array}{c} {\hat{\pi }}_{j}≔X_{j}/n_{1},\;j=1,2,3,\;\;{\hat{p}}_{Y}≔Y/n_{2}. \end{array}$$ (10)

(*X*_1_, *X*_2_, *X*_3_) and *Y* are assumed to belong to independent samples of sizes *n*_1_ [females] and *n*_2_ [males] from a multinomial distribution with parameters (*π*_1_, *π*_2_, *π*_3_) and a binomial distribution with parameter *p*_*Y*_, respectively. Recalling (7) and (8), this leads to the expression

$$\begin{array}{cc} \hat{\Delta } & =[{\left(\text{log}\left({\hat{\pi }}_{2}\right)-\left(\text{log}\left({\hat{\pi }}_{1}\right)+\text{log}\left({\hat{\pi }}_{3}\right)\right)/2-\text{log}\left(2\right)\right)}^{2}+\;\\ & {\left(\text{log}\left({\hat{\pi }}_{1}+{\hat{\pi }}_{2}/2\right)-\text{log}\left(1-{\hat{\pi }}_{1}-{\hat{\pi }}_{2}/2\right)-\text{log}\left({\hat{p}}_{Y}\right)+\text{log}\left(1-{\hat{p}}_{Y}\right)\right)}^{2}{]}^{1/2}. \end{array}$$ (11)

As usual in an asymptotic treatment of inferential procedures for two-sample settings, all statements about convergence in distribution of variables being functions of the ${\hat{\pi }}_{j}$ and ${\hat{p}}_{Y}$ will hold under the assumption that the relative sample sizes *n*_1_/*N* and *n*_2_/*N* tend to nondegenerate limits λ and 1 − λ, say, as the total sample size *N* = *n*_1_ + *n*_2_ increases to infinity. The basic properties of the multinomial family and the independence of (*X*_1_, *X*_2_, *X*_3_) and *Y* ensure that the limiting distribution of $\sqrt{N}(\left({\hat{\pi }}_{1},{\hat{\pi }}_{2},{\hat{\pi }}_{3},{\hat{p}}_{Y},1-{\hat{p}}_{Y}\right)-\left(\pi_{1},\pi_{2},\pi_{3},p_{Y},1-p_{Y}\right))$ is multivariate normal with expected value zero and (singular) covariance matrix

$$\begin{array}{c} \Sigma =(\begin{array}{ccccc} \frac{1}{\lambda }\pi_{1}\left(1-\pi_{1}\right) & -\frac{1}{\lambda }\pi_{1}\pi_{2} & -\frac{1}{\lambda }\pi_{1}\pi_{3} & 0 & 0\\ -\frac{1}{\lambda }\pi_{1}\pi_{2} & \frac{1}{\lambda }\pi_{2}\left(1-\pi_{2}\right) & -\frac{1}{\lambda }\pi_{2}\pi_{3} & 0 & 0\\ -\frac{1}{\lambda }\pi_{1}\pi_{3} & -\frac{1}{\lambda }\pi_{2}\pi_{3} & \frac{1}{\lambda }\pi_{3}\left(1-\pi_{3}\right) & 0 & 0\\ 0 & 0 & 0 & \frac{1}{1-\lambda }p_{Y}\left(1-p_{Y}\right) & -\frac{1}{1-\lambda }p_{Y}\left(1-p_{Y}\right)\\ 0 & 0 & 0 & -\frac{1}{1-\lambda }p_{Y}\left(1-p_{Y}\right) & \frac{1}{1-\lambda }p_{Y}\left(1-p_{Y}\right) \end{array}). \end{array}$$ (12)

Weak convergence of $\sqrt{N}(\left({\hat{\pi }}_{1},{\hat{\pi }}_{2},{\hat{\pi }}_{3},{\hat{p}}_{Y},1-{\hat{p}}_{Y}\right)-(\pi_{1},\pi_{2},\pi_{3},$ *p*_*Y*_, 1 − *p*_*Y*_)) to a centered Gaussian distribution with the above covariance structure is the starting point for establishing the following result (for details of a proof see S1 Appendix).

Proposition 1. Let ${\hat{\Delta }}_{f}^{\pm }$ and ${\hat{\Delta }}_{m}^{\pm }$ denote the plug-in estimators of the parametric functions $\Delta_{f}^{\pm }$ and $\Delta_{m}^{\pm }$. Then, as *N* → ∞, the joint distribution of these estimators centered at their population analogues and scaled by $\sqrt{N}$ converges to the product of two normal distributions with expected value zero and variances given by

$$\begin{array}{c} \sigma_{f;\lambda }^{2}=\frac{1}{\lambda }(\frac{1}{4}\frac{1-\pi_{2}}{\pi_{1}\pi_{3}}+\frac{1}{\pi_{2}}), \end{array}$$ (13)

$$\begin{array}{c} \sigma_{m;\lambda }^{2}=\frac{1}{\left(1-\lambda \right)p_{Y}\left(1-p_{Y}\right)}-\frac{1}{\lambda {\left(1-\pi_{1}-\pi_{2}/2\right)}^{2}}+\;\frac{\pi_{1}+\pi_{2}/4}{\lambda {\left(1+\pi_{2}/2\right)}^{2}{\left(1-\pi_{1}-\pi_{2}/2\right)}^{2}}. \end{array}$$ (14)

The plug-in estimator of the joint distance measure Δ to be eventually used for HWE assessment can be written as $\hat{\Delta }=\sqrt{{\hat{\Delta }}_{f}^{2}+{\hat{\Delta }}_{m}^{2}}$ with ${\hat{\Delta }}_{G}^{2}={\left({\hat{\Delta }}_{G}^{\pm }\right)}^{2}$, *G* ∈ {*f*, *m*}. Hence, except for suitable centering and rescaling by means of $\sqrt{N}$, it has a limiting normal distribution whose variance is a weighted average of $\sigma_{f;\lambda }^{2}$ and $\sigma_{m;\lambda }^{2}$. Precisely speaking, there holds the following

Proposition 2. Assume that for at least one subgroup *G* ∈ {*f*, *m*}, the true value of $\Delta_{G}^{\pm }$ does not vanish. Then, as *N* → ∞, $\sqrt{N}\left(\hat{\Delta }-\Delta \right)$ converges in law to a normally distributed variable with expectation zero and variance given by

$$\begin{array}{c} \tau_{\lambda }^{2}={\left(\Delta_{f}^{2}+\Delta_{m}^{2}\right)}^{-1}(\Delta_{f}^{2}\;\sigma_{f;\lambda }^{2}+\Delta_{m}^{2}\;\sigma_{m;\lambda }^{2})\;. \end{array}$$ (15)

*Proof*. The result follows directly from Proposition 1 by means of the delta method (cf. [22], § 14.6).

Obviously, $\Delta_{f}^{2}$, $\Delta_{m}^{2}$, $\sigma_{f;\lambda }^{2}$ and $\sigma_{m;\lambda }^{2}$ are all continuous functions of (*π*_1_, *π*_2_, *π*_3_, *p*_*Y*_) so that the same holds true for the asymptotic variance $\tau_{\lambda }^{2}$ of $\sqrt{N}\hat{\Delta }$. Since the relative frequencies $\left({\hat{\pi }}_{1},{\hat{\pi }}_{2},{\hat{\pi }}_{3},{\hat{p}}_{Y}\right)$ are consistent for the corresponding population frequencies, this implies, that plugging in the latter in all terms appearing on the right-hand side of Eq (15) and replacing the limiting relative size λ of the sample of females with the actual proportion *n*_1_/(*n*_1_ + *n*_2_) yields a consistent estimator of $\tau_{\lambda }^{2}$. Consistency of this estimator denoted ${\hat{\tau }}_{n_{1},n_{2}}^{2}$ in the sequel, allows us to infer from Proposition 2 that there holds

$$\sqrt{N}\frac{\hat{\Delta }-\Delta }{{\hat{\tau }}_{n_{1},n_{2}}}\overset{L}{\to }Z\sim N(0,1)\;\text{as}\;N\to \infty .$$ (16)

The testing problem which we are interested in reads in formal terms

$$\begin{array}{c} H_{0}:\Delta \geq \varepsilon \;\text{versus}\;H_{1}:\Delta <\varepsilon , \end{array}$$ (17)

and it can be solved through checking an upper confidence bound to the target parameter Δ for non-exceedance of the equivalence margin *ε*. By (16), an upper confidence limit to Δ at asymptotic level 1 − *α* is given by

$$\begin{array}{c} {\bar{\;C}}_{\alpha ;n_{1},n_{2}}^{\Delta }\left({\hat{\pi }}_{1},{\hat{\pi }}_{2},{\hat{\pi }}_{3},{\hat{p}}_{Y}\right)=\hat{\Delta }+z_{1-\alpha }{\hat{\tau }}_{n_{1},n_{2}}/\sqrt{n_{1}+n_{2}}\;. \end{array}$$ (18)

Finally, as the critical region of the corresponding test at asymptotic level *α* for (17), we obtain

$$\begin{array}{c} \begin{array}{c} C_{n_{1},n_{2}}\left(\alpha \right)=\{\left(x_{1},x_{2},x_{3},y\right)|\;0\leq x_{1},x_{2},x_{3}\leq n_{1},\;\\ x_{1}+x_{2}+x_{3}=n_{1},0\leq y\leq n_{2},\;\;{\bar{\;C}}_{\alpha ;n_{1},n_{2}}^{\Delta }\left(x_{1}/n_{1},x_{2}/n_{1},x_{3}/n_{1},y/n_{2}\right)<\varepsilon \}. \end{array} \end{array}$$ (19)

### Exact and approximate methods of computing rejection probabilities and sample-size planning

The rejection probability of the test with critical region (19) under arbitrary parameter configurations is accessible to exact numerical computation. Exploiting the assumed independence of (*X*_1_, *X*_2_, *X*_3_) and *Y*, we can write:

$$\begin{array}{c} \begin{array}{c} P_{\pi ,p_{Y}}[\left(X_{1},X_{2},X_{3},Y\right)\in C_{n_{1},n_{2}}\left(\alpha \right)]=\;\\ \sum_{x_{1}=0}^{n_{1}}\sum_{x_{2}=0}^{n_{1}-x_{1}}\sum_{y=0}^{n_{2}}[\frac{n_{1}!n_{2}!\pi_{1}^{x_{1}}\pi_{2}^{x_{2}}\pi_{3}^{n_{1}-x_{1}-x_{2}}p_{Y}^{y}{\left(1-p_{Y}\right)}^{n_{2}-y}}{x_{1}!x_{2}!\left(n_{1}-x_{1}-x_{2}\right)!y!\left(n_{2}-y\right)!}\;\\ \;\times \;I_{\left(0,\infty \right)}(\varepsilon -{\bar{\;C}}_{\alpha ;n_{1},n_{2}}^{\Delta }(\frac{x_{1}}{n_{1}},\frac{x_{2}}{n_{1}},\frac{n_{1}-x_{1}-x_{2}}{n_{1}},\frac{y}{n_{2}}))], \end{array} \end{array}$$ (20)

where **I**_(0,∞)_(⋅) denotes the indicator of the positive real half-line. Evaluation of the triple sum appearing on the right-hand side of this equation by means of the SAS/IML script we made available for that purpose is fast enough for keeping execution time within reasonable limits even for sample sizes exceeding 1,000.

#### Planning a study under a non-null alternative

Under any alternative $\left(\pi_{1}^{*},\pi_{2}^{*},\pi_{3}^{*},p_{Y}^{*}\right)$, say, for which the assumptions of Proposition 2 are satisfied, an approximate formula for the sample size required in order to guarantee that the power does not fall short of some prespecified target value 1 − *β*, say, is readily obtained. According to that result, the rejection probability of the test using the critical region defined in (19) under an arbitrary parameter configuration with Δ > 0 converges to $\Phi [z_{\alpha }-\sqrt{N}\left(\Delta -\varepsilon \right)/\tau_{\lambda })]$ as *N* → ∞ and *n*_1_/*N* → λ. In terms of Δ, our testing problem is one of one-sided equivalence or, as one would put it in the language of the methodology of clinical trials, of non-superiority. In the literature on asymptotic testing procedures for non-inferiority problems (cf. [23]), it is recommended to approximate the power of an asymptotic test with critical region $\left\{\sqrt{N}\left(T_{N}-\theta_{0}\right)/\sigma_{0}>z_{1-\alpha }\right\}$ through computing the probability that the data fall in this region from a normal distribution with variance $\sigma_{1}^{2}$ rather than $\sigma_{0}^{2}$, where $\sigma_{1}^{2}$ denotes the limiting variance of $\sqrt{N}T_{N}$ under the selected alternative *θ* = *θ*_1_ > *θ*_0_. Adapting this approach in the straightforward way to the setting we are dealing with and denoting the distance of $\left(\Delta_{f}^{\pm },\Delta_{m}^{\pm }\right)$ from zero under the selected alternative by Δ* leads to

$$\begin{array}{c} POW_{\varepsilon }\left(\Delta^{*};\lambda ,N\right)\approx \Phi [({\tilde{\tau }}_{\lambda }\;z_{\alpha }-\sqrt{N}\left(\Delta^{*}-\varepsilon \right))/\tau_{\lambda }]\;. \end{array}$$ (21)

In this approximate equation, $\tau_{\lambda }^{2}$ has to be computed by evaluating (13)–(15) with $\left(\pi_{1},\pi_{2},\pi_{3},p_{Y}\right)=\left(\pi_{1}^{*},\pi_{2}^{*},\pi_{3}^{*},p_{Y}^{*}\right)$, and in order to determine ${\tilde{\tau }}_{\lambda }^{2}$, the same formulas have to be applied with some $\left({\tilde{\pi }}_{1},{\tilde{\pi }}_{2},{\tilde{\pi }}_{3},{\tilde{p}}_{Y}\right)$ such that the corresponding point in the paramter space of $\left(\Delta_{f}^{\pm },\Delta_{m}^{\pm }\right)$ lies on the circle of radius *ε* around the origin. For definiteness, we propose to choose $\left({\tilde{\pi }}_{1},{\tilde{\pi }}_{2},{\tilde{\pi }}_{3},{\tilde{p}}_{Y}\right)$ as conjugate to $\left(\pi_{1}^{*},\pi_{2}^{*},\pi_{3}^{*},p_{Y}^{*}\right)$, in the sense, that we have ${\tilde{\pi }}_{1}=\pi_{1}^{*}$ and ${\tilde{\pi }}_{2}$, ${\tilde{\pi }}_{3}$, ${\tilde{p}}_{Y}$ are determined through solving the equations $\Delta_{f}^{\pm }\left(\tilde{\pi },{\tilde{p}}_{Y}\right)=\varepsilon /\sqrt{2}=\Delta_{m}^{\pm }\left(\tilde{\pi },{\tilde{p}}_{Y}\right)$. The final step required for transforming (21) into the desired sample size formula consists of specifying the power 1 − *β* one wants to attain and solving the equation $\Phi [({\tilde{\tau }}_{\lambda }z_{\alpha }-\sqrt{N}\left(\Delta^{*}-\varepsilon \right))/\tau_{\lambda }]=1-\beta$ for *N* which yields after a little algebra the expression

$$\begin{array}{c} N=\frac{{\left({\tilde{\tau }}_{\lambda }z_{1-\alpha }+\tau_{\lambda }z_{1-\beta }\right)}^{2}}{{\left(\Delta^{*}-\varepsilon \right)}^{2}}\;. \end{array}$$ (22)

#### The case of null alternatives

Despite the often unsatisfactory accuracy provided by formula (22) for sample size planning under non-null alternatives, its derivability from standard weak convergence results is obvious. In contrast, for the power of the test with critical region (19) under alternatives under which the true value of Δ is zero, no useful approximation by means of a simple Gaussian distribution exists. An approach which will turn out to solve the problem in a very satisfactory way is based on the following concept.

Definition 1. Let *Z*_1_…, *Z*_*k*_ be mutually independent with $Z_{j}∼N\left(0,c_{j}^{2}\right)$ where *c*_1_ = 1 and *c*_*j*_ denotes an arbitrary positive constant for all *j* = 2, …, *k*. Then, the distribution of $Q≔\sqrt{\sum_{j=1}^{k}Z_{j}^{2}}$ is called an extended *χ*-distribution with *k* degrees of freedom and parameter **c**. Its cdf (cumulative distribution function) will be written $Q_{c}\left(\cdot \right)$.

Although $Q_{c}\left(\cdot \right)$ is not a known statistical function for which the packages provide predefined routines (except, of course, for the standard *χ*-distribution corresponding to the special case *c*_*j*_ = 1∀*j* = 1, …, *k*), it is not difficult to find a representation which can serve as a basis for an easy to implement algorithm for numerical computation. In the special case *k* = 2 where we drop the subscript from the only non-unity component of **c**, we can rely on the following result.

Lemma 1. For arbitrarily fixed *c* > 0 and any $q\in R_{+}$, there holds

$$\begin{array}{c} Q_{c}\left(q\right)=2\int_{-q}^{q}\Phi (\frac{1}{c}\sqrt{q^{2}-z_{1}^{2}})\phi \left(z_{1}\right)dz_{1}-[2\Phi (q)-1], \end{array}$$ (23)

with *ϕ*(⋅) and *Φ*(⋅) denoting, as usual, the standard normal density and cdf, respectively.

*Proof*. See S2 Appendix.

The key computational tool being required for working with the distribution function $Q_{c}\left(\cdot \right)$ in practice is an efficient procedure for the evaluation of the integral appearing on the right-hand side of Eq (23). The SAS/IML script we developed for that purpose uses Gauss-Legendre 96-point quadrature and partitioning of the range of integration into 10 subintervals. Even when numerical integration is done at that high level of accuracy, the algorithm is still fast enough to enable also exact computation of the corresponding quantile function $Q_{c}^{-1}\left(\cdot \right)$. The relevance of the distribution function $Q_{c}\left(\cdot \right)$ for finding an approximation to the power of our test for goodness of fit to HWE becomes obvious from

Proposition 3. Let $P_{*}^{\left(N\right)}\left(\;\cdot \;\right)$ denote the joint distribution of (*X*_1_, *X*_2_, *X*_3_, *Y*) under some fixed parameter configuration $\left(\pi_{1}^{*},\pi_{2}^{*},\pi_{3}^{*},\pi_{Y}^{*}\right)$ with $\Delta_{f}^{\pm }=0=\Delta_{m}^{\pm }$. Then, there holds for every *d* > 0

$$\begin{array}{c} P_{*}^{\left(N\right)}[\sqrt{N}\hat{\Delta }\leq d]\to Q_{\sigma_{m;\lambda }/\sigma_{f;\lambda }}(d/\sigma_{f;\lambda })\;\text{as}\;N\to \infty .\; \end{array}$$ (24)

*Proof*. From the definition of $\hat{\Delta }$, it is immediately clear that denoting the Euclidean distance of any point (*z*_1_, *z*_2_) in the plane from the origin by *q*(*z*_1_, *z*_2_), we can write

$$\begin{array}{c} \sqrt{N}\hat{\Delta }=\sigma_{f;\lambda }\;q(\sqrt{N}{\hat{\Delta }}_{f}^{\pm }/\sigma_{f;\lambda },\left(\sigma_{m;\lambda }/\sigma_{f;\lambda }\right)\sqrt{N}{\hat{\Delta }}_{m}^{\pm }/\sigma_{m;\lambda })\;. \end{array}$$ (25)

Furthermore, by Proposition 1, we know that in the case of $\Delta_{G}^{\pm }$ vanishing both for *G* = *f* and *G* = *m*, there holds

$$\begin{array}{c} (\sqrt{N}{\hat{\Delta }}_{f}^{\pm }/\sigma_{f;\lambda },\left(\sigma_{m;\lambda }/\sigma_{f;\lambda }\right)\sqrt{N}{\hat{\Delta }}_{m}^{\pm }/\sigma_{m;\lambda })\overset{L}{\to }(Z_{1},Z_{2})\;\text{as}\;N\to \infty \;, \end{array}$$ (26)

where (*Z*_1_, *Z*_2_) are as assumed in Definition 1 with *k* = 2, *c*_2_ = *σ*_*m*;λ_/*σ*_*f*;λ_. Since *q*(⋅, ⋅) is continuous, the mapping theorem for weakly convergent sequences of probability measures (see, e.g., [24], p. 379) allows us to conclude from (26) that we also have

$$\begin{array}{c} q(\sqrt{N}{\hat{\Delta }}_{f}^{\pm }/\sigma_{f;\lambda },\left(\sigma_{m;\lambda }/\sigma_{f;\lambda }\right)\sqrt{N}{\hat{\Delta }}_{m}^{\pm }/\sigma_{m;\lambda })\overset{L}{\to }q\left(Z_{1},Z_{2}\right)∼Q_{\sigma_{m;\lambda }/\sigma_{f;\lambda }}\left(\cdot \right)\;\text{as}\;N\to \infty , \end{array}$$

which in view of (25) completes the proof.

The steps to be taken in order to exploit Proposition 3 for approximating the probability of the event $\left\{\hat{\Delta }+z_{1-\alpha }{\hat{\tau }}_{n_{1},n_{2}}/\sqrt{N}<\varepsilon \right\}$ under a fixed null alternative $\left(\pi_{1}^{*},\pi_{2}^{*},\pi_{3}^{*},\pi_{Y}^{*}\right)$ are analogous to those which lead from Proposition 2 to the power approximation (21) for the case of non-null alternatives. First of all, we replace the empirical asymptotic standard error ${\hat{\tau }}_{n_{1},n_{2}}$ of $\sqrt{N}\hat{\Delta }$ with ${\tilde{\tau }}_{\lambda }$, i.e., the square root of the theoretical asymptotic variance of $\sqrt{N}\hat{\Delta }$ computed at a point $\left({\tilde{\pi }}_{1},{\tilde{\pi }}_{2},{\tilde{\pi }}_{3},{\tilde{p}}_{Y}\right)$ on the boundary of the equivalence circle in the parameter space of $\left(\Delta_{f}^{\pm },\Delta_{m}^{\pm }\right)$ being conjugate to $\left(\pi_{1}^{*},\pi_{2}^{*},\pi_{3}^{*},p_{Y}^{*}\right)$ in the sense made explicit above (→ paragraph following Eq 19). Making this substitution reduces the problem of power computation against null alternatives to that of calculating

$$\begin{array}{c} P_{*}^{\left(N\right)}[\sqrt{N}\hat{\Delta }<\sqrt{N}\varepsilon -z_{1-\alpha }{\tilde{\tau }}_{\lambda }]\approx Q_{\sigma_{m;\lambda }/\sigma_{f;\lambda }}([\sqrt{N}\varepsilon -z_{1-\alpha }{\tilde{\tau }}_{\lambda }]/\sigma_{f;\lambda })\;. \end{array}$$ (27)

By definition (recall Eq 13), ${\tilde{\tau }}_{\lambda }^{2}$ is a weighted mean of ${\tilde{\sigma }}_{f;\lambda }^{2}$ and ${\tilde{\sigma }}_{m;\lambda }^{2}$, with ${\tilde{\sigma }}_{f;\lambda }^{2}$ and ${\tilde{\sigma }}_{m;\lambda }^{2}$ denoting the asymptotic variance of $\sqrt{N}{\hat{\Delta }}_{G}^{\pm }$ and $\sqrt{N}{\hat{\Delta }}_{m}^{\pm }$ computed by plugging-in $\left({\tilde{\pi }}_{1},\tilde{\pi },{\tilde{\pi }}_{3},{\tilde{p}}_{Y}\right)$ in (13) and (14), respectively. Preliminary numerical investigations have clearly shown that the accuracy of the power approximation (27) can be considerably improved through replacing ${\tilde{\tau }}_{\lambda }^{2}$ with $\max\{{\tilde{\sigma }}_{f;\lambda }^{2},{\tilde{\sigma }}_{m;\lambda }^{2}\}$. Finally, solving the equation $Q_{\sigma_{m;\lambda }/\sigma_{f;\lambda }}([\sqrt{N}\varepsilon -z_{1-\alpha }\max\left\{{\tilde{\sigma }}_{f;\lambda },{\tilde{\sigma }}_{m;\lambda }\right\}]/\sigma_{f;\lambda })=1-\beta$ for *N* yields

$$\begin{array}{c} N=\frac{{\left(z_{1-\alpha }\max\left\{{\tilde{\sigma }}_{f;\lambda },{\tilde{\sigma }}_{m;\lambda }\right\}+\sigma_{f;\lambda }Q_{\sigma_{m;\lambda }/\sigma_{f;\lambda }}^{-1}\left(1-\beta \right)\right)}^{2}}{\varepsilon^{2}} \end{array}$$ (28)

as the desired null-alternative analogue of (22)

## Results

### Small-sample properties of the proposed test for goodness of fit

**Fig 2:**
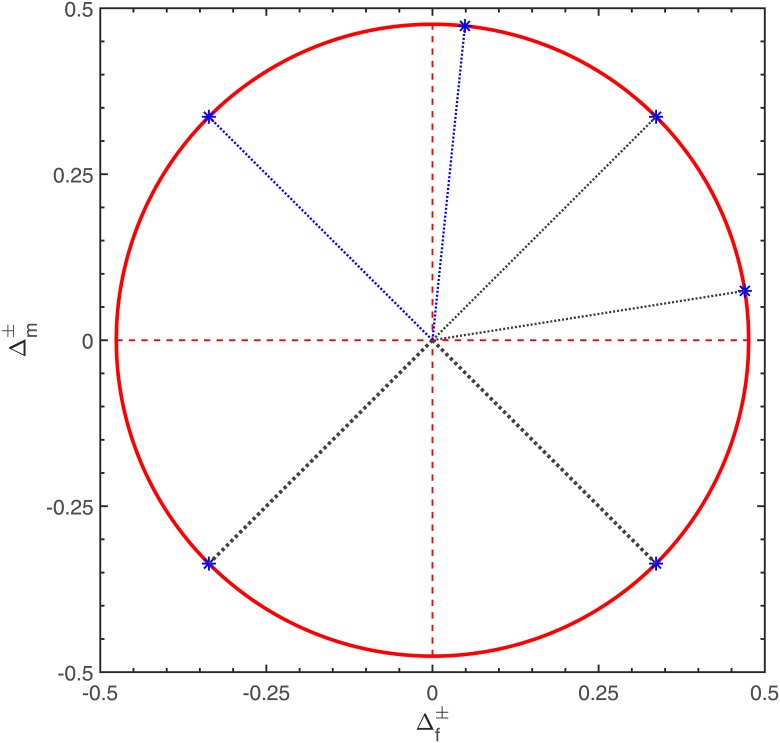
Visualization of the parameter configurations covered by Table 1 as points in the $\left(\Delta_{f}^{\pm },\Delta_{m}^{\pm }\right)$-plane.

A first basic question to answer is whether the procedure maintains the nominal significance level when performed with samples of sizes being commonly available in genetic association studies. The results shown in Tables 1 and 2 give the exact rejection probabilities at a selection of points in the parameter space lying on the common boundary of the hypotheses we are interested in. The position of these points in the $\left(\Delta_{f}^{\pm },\Delta_{m}^{\pm }\right)$-plane is shown in Fig 2. In the constellations covered by Tables 1 and 2 and many other instances we found no single case of an anti-conservative behavior. On the other hand, it becomes obvious from the entries in the table that the convergence of the rejection probability under the null hypothesis of relevant deviations from HWE to the nominal significance level is comparatively slow. Even for settings with sample sizes of more than 1000 in both subgroups, the absolute difference by which the rejection probability under *H*_0_ falls below the nominal level of 5% can still be larger than 1%.

**Table 1:** Exact rejection probabilities of the goodness-of-fit test with critical region (19) at the common boundary of the hypotheses (17). [Nominal significance level *α* = 0.05; equivalence margin $\varepsilon =\sqrt{2}\text{log}\left(1.4\right)\approx 0.48$].

*π*_1_	*π*_2_	$p_{{}_{X}}^{†)}$	*p*_*Y*_	$\Delta_{f}^{\pm }$	$\Delta_{m}^{\pm }$	*n*_1_	*n*_2_	Rej. Prob.
0.25	0.57897	0.53949	0.45557	0.33647	0.33647	100	100	0.01103
″	″	″	″	″	″	400	400	0.03505
″	″	″	″	″	″	400	600	0.03474
″	″	″	″	″	″	600	400	0.03966
″	″	″	″	″	″	800	800	0.03972
″	″	″	″	″	″	1200	1200	0.04164
0.25	0.41402	0.45701	0.37546	−0.33647	0.33647	100	100	0.01381
″	″	″	″	″	″	400	400	0.03944
″	″	″	″	″	″	400	600	0.03906
″	″	″	″	″	″	600	400	0.04352
″	″	″	″	″	″	800	800	0.04316
″	″	″	″	″	″	1200	1200	0.04459
0.25	0.57897	0.53949	0.62123	0.33647	−0.33647	100	100	0.01167
″	″	″	″	″	″	400	400	0.03475
″	″	″	″	″	″	400	600	0.03432
″	″	″	″	″	″	600	400	0.03963
″	″	″	″	″	″	800	800	0.03955
″	″	″	″	″	″	1200	1200	0.04154
0.25	0.41402	0.45701	0.54093	−0.33647	−0.33647	100	100	0.01289
″	″	″	″	″	″	400	400	0.03948
″	″	″	″	″	″	400	600	0.03944
″	″	″	″	″	″	600	400	0.04356
″	″	″	″	″	″	800	800	0.04326
″	″	″	″	″	″	1200	1200	0.04467
0.25	0.51212	0.50606	0.38958	0.04879	0.47334	100	100	0.01634
″	″	″	″	″	″	400	400	0.04023
″	″	″	″	″	″	400	600	0.03830
″	″	″	″	″	″	600	400	0.04380
″	″	″	″	″	″	800	800	0.04323
″	″	″	″	″	″	1200	1200	0.04452
0.25	0.60637	0.55319	0.53475	0.47000	0.07432	100	100	0.00912
″	″	″	″	″	″	400	400	0.03175
″	″	″	″	″	″	400	600	0.03538
″	″	″	″	″	″	600	400	0.03145
″	″	″	″	″	″	800	800	0.03748
″	″	″	″	″	″	1200	1200	0.03989
0.09	0.52274	0.35137	0.27899	0.33647	0.33647	100	100	0.00702
″	″	″	″	″	″	400	400	0.03240
″	″	″	″	″	″	400	600	0.03282
″	″	″	″	″	″	600	400	0.03705
″	″	″	″	″	″	800	800	0.03785
″	″	″	″	″	″	1200	1200	0.04012
0.09	0.32718	0.25359	0.19529	−0.33647	0.33647	100	100	0.00041
″	″	″	″	″	″	400	400	0.03403
″	″	″	″	″	″	400	600	0.03509
″	″	″	″	″	″	600	400	0.03873
″	″	″	″	″	″	800	800	0.04017
″	″	″	″	″	″	1200	1200	0.04235

^†)^ = *π*_1_ + *π*_2_/2 [≡ allele frequency among females]

**Table 2:** Exact rejection probabilities of the goodness-of-fit test with critical region (19) at additional points on the common boundary of the hypotheses (17). [Nominal significance level *α* = 0.05; equivalence margin $\varepsilon =\sqrt{2}\text{log}(1.4)\approx 0.48$].

*π*_1_	*π*_2_	$p_{{}_{X}}^{†)}$	*p*_*Y*_	$\Delta_{f}^{\pm }$	$\Delta_{m}^{\pm }$	*n*_1_	*n*_2_	Rej. Prob.
0.09	0.52274	0.35137	0.43130	0.33647	−0.33647	100	100	0.00941
″	″	″	″	″	″	400	400	0.03367
″	″	″	″	″	″	400	600	0.03414
″	″	″	″	″	″	600	400	0.03765
″	″	″	″	″	″	800	800	0.03863
″	″	″	″	″	″	1200	1200	0.04079
0.09	0.32718	0.25359	0.32233	−0.33647	−0.33647	100	100	0.00325
″	″	″	″	″	″	400	400	0.03639
″	″	″	″	″	″	400	600	0.03746
″	″	″	″	″	″	600	400	0.03976
″	″	″	″	″	″	800	800	0.04140
″	″	″	″	″	″	1200	1200	0.04326
0.09	0.43445	0.30723	0.21645	0.04879	0.47334	100	100	0.00347
″	″	″	″	″	″	400	400	0.03657
″	″	″	″	″	″	400	600	0.03401
″	″	″	″	″	″	600	400	0.04222
″	″	″	″	″	″	800	800	0.04137
″	″	″	″	″	″	1200	1200	0.04012
0.09	0.56438	0.37219	0.35500	0.47000	0.07432	100	100	0.00762
″	″	″	″	″	″	400	400	0.03177
″	″	″	″	″	″	400	600	0.03555
″	″	″	″	″	″	600	400	0.03152
″	″	″	″	″	″	800	800	0.03759
″	″	″	″	″	″	1200	1200	0.04003
0.00564	0.18874	0.10001	0.07354	0.33647	0.33647	400	400	0.00726
″	″	″	″	″	″	800	800	0.02603
″	″	″	″	″	″	1200	1200	0.03304
″	″	″	″	″	″	1600	1600	0.03643

^†)^ = *π*_1_ + *π*_2_/2 [≡ allele frequency among females]

In testing for goodness rather than lack of fit of empirical distributions to some probability model, the specific alternatives of primary interest are usually those satisfying the null hypothesis of the corresponding test for lack of fit. In the present case, under such null alternatives, there is only a single free parameter left, namely, the common frequency of allele A in females and males. As becomes obvious from the results shown in Table 3, the power of the proposed test against null alternatives is highly sensitive to changes in this parameter. For an allele frequency of 50%, 400 observations from each subpopulation are sufficient to increase the power above 95%. In contrast, for alleles occurring at a frequency of only 10% both in females and males, the sample size per group must be a bit more than three times as large if one wants to rule out that the power drops below 75%.

**Table 3:** Exact power of the goodness-of-fit test with critical region (19) under selected null alternatives. [Nominal significance level *α* = 0.05; equivalence margin $\varepsilon =\sqrt{2}\text{log}\left(1.4\right)\approx 0.48$].

*π*_1_	*π*_2_	$p_{{}_{X}}^{†)}$	*p*_*Y*_	$\Delta_{f}^{\pm }$	$\Delta_{m}^{\pm }$	*n*_1_	*n*_2_	Rej. Prob.
0.25	0.5	0.5	0.5	0.00	0.00	100	100	0.10239
″	″	″	″	″	″	400	400	0.95828
″	″	″	″	″	″	400	600	0.98461
″	″	″	″	″	″	600	400	0.98224
″	″	″	″	″	″	800	800	0.99979
″	″	″	″	″	″	1200	1200	1.00000
0.16	0.48	0.4	0.4	0.00	0.00	100	100	0.09111
″	″	″	″	″	″	400	400	0.94585
″	″	″	″	″	″	400	600	0.97755
″	″	″	″	″	″	600	400	0.97700
″	″	″	″	″	″	800	800	0.99965
″	″	″	″	″	″	1200	1200	1.00000
0.09	0.42	0.3	0.3	0.00	0.00	100	100	0.03006
″	″	″	″	″	″	400	400	0.88172
″	″	″	″	″	″	400	600	0.93296
″	″	″	″	″	″	600	400	0.94927
″	″	″	″	″	″	800	800	0.99818
″	″	″	″	″	″	1200	1200	0.99998
0.04	0.32	0.2	0.2	0.00	0.00	100	100	0.00008
″	″	″	″	″	″	400	400	0.62410
″	″	″	″	″	″	400	600	0.70607
″	″	″	″	″	″	600	400	0.79884
″	″	″	″	″	″	800	800	0.96535
″	″	″	″	″	″	1200	1200	0.99750
0.01	0.18	0.1	0.1	0.00	0.00	100	100	0.00000
″	″	″	″	″	″	400	400	0.06914
″	″	″	″	″	″	400	600	0.10738
″	″	″	″	″	″	600	400	0.18311
″	″	″	″	″	″	800	800	0.45744
″	″	″	″	″	″	1200	1200	0.73707

^†)^ = *π*_1_ + *π*_2_/2 [≡ allele frequency among females]

With regard to power, null alternatives are obviously most favorable parameter configurations, and perfect fit of the distributions underlying the data to the model is of course a limiting case which will hardly occur in reality. Given anything else, it has to be expected that the power drops quite rapidly when the point in the parameter plane of $\left(\Delta_{f}^{\pm },\Delta_{m}^{\pm }\right)$ corresponding to the specific alternative of interest is shifted from the origin towards the boundary of the equivalence circle. In order to obtain insight in the speed of this decline in power, we calculated the exact power of the test at nominal level *α* = 0.05 attained at 9 equidistant points on the segment between 0 and $\varepsilon =\sqrt{2}\text{log}\left(1.4\right)$ on the main diagonal for samples of size 800 each. From the results of these calculations which are shown in Table 4, one can see that increasing the deviation from perfect HWE by 50% of the equivalence margin set under the alternative hypothesis to be established decreases the exact power of the proposed test from over 95% to 60.7%.

**Table 4:** Exact power of the goodness-of-fit test with critical region (19) and 800 observations per subgroup under alternatives specifying that the true deviation from HWE is a non-zero fraction of that considered compatible with equivalence. [Nominal significance level *α* = 0.05; equivalence margin $\varepsilon =\sqrt{2}\text{log}\left(1.4\right)\approx 0.48$].

*π*_1_	*π*_2_	$p_{{}_{X}}^{†)}$	*p*_*Y*_	$\Delta_{f}^{\pm }$	$\Delta_{m}^{\pm }$	Δ	Rej. Prob.
0.04	0.32870	0.20435	0.19893	0.03365	0.03365	0.04758	0.94943
″	0.33755	0.20878	0.19788	0.06729	0.06729	0.09517	0.91198
″	0.34656	0.21328	0.19684	0.10094	0.10094	0.14275	0.84553
″	0.35573	0.21787	0.19580	0.13459	0.13459	0.19034	0.74342
″	0.36506	0.22253	0.19478	0.16824	0.16824	0.23792	0.60695
″	0.37453	0.22727	0.19377	0.20188	0.20188	0.28551	0.45004
″	0.38415	0.23208	0.19276	0.23553	0.23553	0.33309	0.29666
″	0.39392	0.23696	0.19176	0.26918	0.26918	0.38068	0.17046
″	0.40382	0.24191	0.19076	0.30283	0.30283	0.42826	0.08390

^†)^ = *π*_1_ + *π*_2_/2 [≡ allele frequency among females]

### Illustrating examples

To illustrate our method, we use the same data as Graffelman and Weir [7]. They illustrated the application of their combined *χ*^2^-test for lack of fit of an X-chromosomal SNP to HWE. The genotype and allele frequencies observed in these examples were extracted from the publicly accessible [25] database of the GENEVA venous thrombosis project, a genomewide association study (GWAS) performed in 2010/11 with the objective to identify genetic variants associated with venous thromboembolism (VTE). The subjects recruited for the project were 1300 VTE cases and 1300 unrelated controls, frequency-matched on 5 elementary criteria. The observed genotype and allele counts obtained in the GENEVA project for four X-chromosomal SNPs (indexed here for brevity by integer numbers) analyzed by [7] are the entries in the left-hand columns of Table 5, which additionally shows the values of the basic estimators required for carrying out the goodness-of-fit test derived in this paper. Except for rs12010339, the upper 95% confidence bound to the combined distance measure Δ falls below the proposed numerical value of the equivalence limit *ε* to Δ so that 3 out of the 4 SNPs under consideration pass the check for approximate compatibility with HWE. The only setting for which there is full coincidence in terms of the qualitative conclusions between our procedure and the lack-of-fit test proposed by [7] is that of rs5968922: with these data, the latter gives a (2-sided) p-value of 100% and thus clearly indicates nonexistence of deviations from HWE. In the other cases, a well-judged synoptic interpretation of the results of both testing procedures requires to take into account that a small p-value of a test tailored for detecting differences in no way rules out that an equivalence test carried out with the same data likewise leads to a positive decision. This follows from the fact that the indifference zone corresponding to the alternative hypothesis of an equivalence problem consists of points which also belong to the alternative to the classical null hypothesis of perfect coincidence with the model. Thus, there are parameter constellations under which both tests may have moderate or even high power (In the setting of Fig 2 this holds true for all interior points of the circular disc with radius *ε* = 2log(1.4)).

**Table 5:** Testing four X-chromosomal SNPs ascertained in the GENEVA project [25] for goodness of fit to HWE. [Nominal significance level *α* = 0.05; equivalence margin $\varepsilon =\sqrt{2}\text{log}\left(1.4\right)\approx 0.48$; decision = “+” ⇔ rejection of the null hypothesis of relevant deviations from HWE. The results for rs12010339 were calculated replacing both zero entries by 1 and decreasing *x*_1_ by 2, in line with common rules for the analysis of sparse contingency tables].

SNP#	*n*_1_	*x*_1_	*x*_2_	*x*_3_	*n*_2_	*y*	$\hat{\Delta }$	${\hat{\tau }}_{n_{1},n_{2}}^{2}$	${\bar{\;C}}_{\alpha ;n_{1},n_{2}}^{\Delta }$	Decision
rs6646338	651	230	314	107	604	399	0.2835	13.2641	0.4526	+
rs12010339	″	651	0	0	605	603	3.9475	157.4547	5.7856	−
rs5935567	″	231	337	83	605	372	0.1964	8.8719	0.3346	+
rs5968922	″	275	296	80	604	392	0.0040	12.1492	0.1658	+

In order to have a broader basis for comparing the new testing procedure with the inverted traditional *χ*^2^-test for lack of fit, we generated by simulation 100,000 samples of varying sizes consisting of genotype distributions observed at an X-chromosomal SNP in a population with pre-specified parameters, and computed the rejection rates of both procedures. For the first half of these simulations, the parameters were chosen as in the 7th horizontal block of Table 1 studying the behavior of the tests under a configuration belonging to the null hypothesis of relevant deviations from HWE. For each individual sample, in the first and second of these simulation experiments, the number of genotyped subjects was chosen to be for both females and males 100 and 1200, respectively. The rejection rates obtained with these data are shown in the upper half of Table 6. The other part of the simulation experiments whose results are summarized in Table 6, were run to compare both procedures in terms of the power against null alternatives generating the data under the parameter configuration appearing in the middle block of Table 3. Not surprisingly, the outcome of comparisons of that kind highly depends on the sample size: for small sample sizes, inverting the lack-of-fit test in the naive way entails gross eceedances of the target significance level, whereas in large samples, the same procedure becomes grossly overconservative. In the latter case, the power falls distinctly below that of the correct goodness-of-fit test, in the former it provides a strong pseudo-advantage in power. Another inherent feature of the inverted lack-of-fit test becoming conspicuous from the entries in Table 6 is that its power against null alternatives, is constant (except for minor deviances due to the large-sample approximations involved) rather than increasing in the sample sizes. Thus, it is lacking a property to be required of any statistical decision procedure which merits being called a test of significance.

**Table 6:** Comparisons between the goodness-of-fit test and the inverted *χ*^2^-test in data sets generated by simulation from a population satisfying the null hypothesis of relevant disequilibrium [upper lines] and being in perfect HWE [lines 3-4], respectively. [Nominal significance level *α* = 0.05; equivalence margin $\varepsilon =\sqrt{2}\text{log}\left(1.4\right)\approx 0.48$; 100,000 replications per Monte Carlo experiment].

*π*_1_	*π*_2_	$p_{{}_{X}}^{†)}$	*p*_*Y*_	$\Delta_{f}^{\pm }$	$\Delta_{m}^{\pm }$	*n*_1_	*n*_2_	Sim. Rej. Prob.		Prop. of concord. dec.
								Gof-Test	invtd. *χ*^2^	
0.09	0.52274	0.35137	0.27899	0.33647	0.33647	100	100	0.00696	0.60024	0.40672
″	″	″	″	″	″	1200	1200	0.03983	0.00000	0.96017
0.09	0.42	0.3	0.3	0.00	0.00	100	100	0.03102	0.95177	0.07925
″	″	″	″	″	″	1200	1200	0.99996	0.94926	0.94930

^†)^ = *π*_1_ + *π*_2_/2 [≡ allele frequency among females]

### Sample size calculation for the test for goodness of fit to HWE

The sample sizes shown in Table 7 as entries in Column 2 and 3 from right are obtained by applying formula (22) to a selection of specific non-null alternatives, again for a nominal significance level of 5% and with the equivalence margin *ε* chosen as proposed in Subsection 2 of M&M. Comparing the exact power attained with these approximate sample sizes which is shown in the right-most column of the table, with the target power of 80% reveals that the accuracy of the approximation is acceptable for settings for which it has to be expected that the number of male subjects is a multiple of the size of the sample of females. In strongly unbalanced cases of the complementary kind, the approximation error becomes much too large for making formula (22) useful for real applications. Even when *n*_2_ has to be much larger than *n*_1_, using (22) for sample size planning of a study where interest is in controlling the power against a non-null alternative leads to marked underestimation of the exact numbers of subjects.

**Table 7:** Sample-sizes approximated by means of formula (22) and exact power effectively attained with them against selected non-null alternatives of the form considered in Table 3. [Nominal significance level *α* = 0.05; target power = 80%; equivalence margin $\varepsilon =\sqrt{2}\;\text{log}(1.4)\approx 0.48$].

*π*_1_	*π*_2_	$p_{{}_{X}}^{†)}$	*p*_*Y*_	$\Delta_{f}^{\pm }$	$\Delta_{m}^{\pm }$	Δ	λ	*n*_1_	*n*_2_	*POW*_*ex*_
0.25	0.54097	0.52048	0.47846	0.16824	0.16824	0.23792	$\frac{1}{2}$	485	485	0.69063
″	″	″	″	″	″	″	$\frac{1}{3}$	449	898	0.75536
″	″	″	″	″	″	″	$\frac{2}{3}$	552	276	0.60427
″	″	″	″	″	″	″	$\frac{1}{4}$	436	1308	0.77938
″	″	″	″	″	″	″	$\frac{3}{4}$	612	204	0.54963
0.04	0.35573	0.21787	0.19580	0.13459	0.13459	0.19034	$\frac{1}{2}$	697	697	0.67720
″	″	″	″	″	″	″	$\frac{1}{3}$	656	1312	0.73626
″	″	″	″	″	″	″	$\frac{2}{3}$	774	387	0.58844
″	″	″	″	″	″	″	$\frac{1}{4}$	641	1923	0.75422
″	″	″	″	″	″	″	$\frac{3}{4}$	846	282	0.53012

^†)^ = *π*_1_ + *π*_2_/2 [≡ allele frequency among females]

Evaluation of the accuracy provided by formula (28) was performed along the same lines as in assessing formula (22): For a selection of null alternatives $\left(\pi_{1}^{*},\pi_{2}^{*},\pi_{3}^{*},p_{Y}^{*}\right)$ and values of the proportion λ of females among all subjects to be recruited, the target power was compared with the exact power attained with the sample sizes required according to the approximation formula. Overall, the results of these comparisons being shown in Table 8 are distinctly more satisfactory than those obtained with formula (22) for alternatives which, in terms of the distance measure Δ, fall in between zero and the equivalence margin *ε*. Except for the low-power settings with 1 − *β* = 0.60, which are of limited relevance for real applications, the maximum of the absolute difference between exact and target power taken over all parameter configurations covered by the table, is less than 3%. More often than not, the solution obtained by means of the formula turns out conservative, in the sense of (slightly) overestimating the sample sizes effectively required.

**Table 8:** Sample-sizes approximated by means of formula (28) and exact power effectively attained with them against selected alternatives exactly satisfying the HWE condition. [Nominal significance level *α* = 0.05; equivalence margin $\varepsilon =\sqrt{2}\;\text{log}(1.4)\approx 0.48$].

$\pi_{1}^{*}$	$\pi_{2}^{*}$	$\pi_{3}^{*}$	$p_{Y}^{*}$	λ	*σ*_*m*;λ_/*σ*_*f*;λ_	1 − *β*	*n*_1_	*n*_2_	*POW*_*ex*_
0.25	0.50	0.25	0.5	1/2	1.22475	0.60	213	213	0.64299
″	″	″	″	″	″	0.80	279	279	0.82359
″	″	″	″	″	″	0.90	338	338	0.91139
″	″	″	″	1/3	1.00000	0.60	163	326	0.61533
″	″	″	″	″	″	0.80	214	428	0.80902
″	″	″	″	″	″	0.90	260	520	0.90585
″	″	″	″	1/4	0.91287	0.60	157	471	0.64940
″	″	″	″	″	″	0.80	205	615	0.82969
″	″	″	″	″	″	0.90	247	741	0.91558
0.09	0.42	0.49	0.3	1/2	1.12250	0.60	264	264	0.61656
″	″	″	″	″	″	0.80	346	346	0.80669
″	″	″	″	″	″	0.90	419	419	0.90118
″	″	″	″	1/3	0.91652	0.60	210	420	0.59908
″	″	″	″	″	″	0.80	276	552	0.79392
″	″	″	″	″	″	0.90	335	670	0.89315
″	″	″	″	1/4	0.83666	0.60	202	606	0.62698
″	″	″	″	″	″	0.80	265	795	0.80995
″	″	″	″	″	″	0.90	321	963	0.90049
0.01	0.18	0.81	0.1	1/2	0.73485	0.60	1054	1054	0.65511
″	″	″	″	″	″	0.80	1375	1375	0.80985
″	″	″	″	″	″	0.90	1668	1668	0.88892
″	″	″	″	1/3	0.60000	0.60	986	1972	0.66802
″	″	″	″	″	″	0.80	1288	2576	0.80764
″	″	″	″	″	″	0.90	1575	3150	0.88403
″	″	″	″	1/4	0.54772	0.60	960	2880	0.66463
″	″	″	″	″	″	0.80	1259	3777	0.80283
″	″	″	″	″	″	0.90	1547	4641	0.88074

## Discussion

It was demonstrated over a decade ago that autosomal SNPs could be tested for HWE in a way being logically adapted to the needs of genetic association studies. It has never been explicitly challenged that this requires to treat goodness rather than lack of fit to the model as the hypothesis to be established. The equivalence test to be performed for establishing goodness of fit has been made available both as an exact optimal procedure [15] and an asymptotic procedure being particularly attractive for practitioners for its computational simplicity [17]. Nevertheless, the process of revising the practice of genetic association studies through switching from lack-of-fit to goodness-of-fit testing in the HWE-related part of preliminary data analysis has taken place only hesitantly up to now.

The problem of extending HWE testing to X-chromosomal SNPs has been addressed in the literature only recently, and the authors of the pertinent papers [7, 10, 11, 13] adopt the traditional perspective treating the statement that the distribution underlying the data satisfies the model, as the null hypothesis.

As is generally the case in the derivation of equivalence testing methods, we had to start with making precise the notion of “sufficient closeness” between the true and the HWE-conforming joint distribution of the genotype frequencies for females and the allele frequency in the subpopulation of males through defining a suitable distance measure. This was done in two steps: First, we introduced separate distance measures for the trinomial genotype distribution among females and the binomial distribution of the count of the allele of interest (denoted by A) among male subjects. Considering the female subpopulation only, the problem of measuring the amount of disequilibrium is the same as in the case of an autosomal diallelic marker. In the existing literature on the latter convincing arguments can be found for looking at the deviation of the relative excess heterozygosity (REH), defined as 1/2 times the frequency of heterozygotes over the geometric mean of the population frequencies of both homozygotic genotypes, from unity. To avoid technical difficulties entailed with distributional parameters with bounded range, we replaced REH with its logarithm throughout. Our proposal to measure the distance between the two binomial distributions involved in terms of the log-odds ratio between the probabilities of obtaining an A-allele in the corresponding subpopulations is in line with the general methodology of equivalence testing. The second step to be taken in order to get a basis for formalizing the notion of approximate compatibility of an X-chromosomal SNP with HWE consisted of selecting a metric on the parameter space of (log *REH*, log *OR*). The most natural candidates for that purpose are Euclidean and Chebyshev distance on $R^{2}$ defined in the usual way, namely by (i) $D_{\text{EUCL}}\left(\left(x_{1}^{\prime },x_{2}^{\prime }\right),\left(x_{1}^{\prime \prime },x_{2}^{\prime \prime }\right)\right)=\sqrt{{\left(x_{1}^{\prime }-x_{1}^{\prime \prime }\right)}^{2}+{\left(x_{2}^{\prime }-x_{2}^{\prime \prime }\right)}^{2}}$ and (ii) $D_{\text{CHEB}}\left(\left(x_{1}^{\prime },x_{2}^{\prime }\right),\left(x_{1}^{\prime \prime },x_{2}^{\prime \prime }\right)\right)=\max|x_{1}^{\prime }-x_{1}^{\prime \prime }\left|,\right|x_{2}^{\prime }-x_{2}^{\prime \prime }|$, respectively. Our preference in favor of option (i) has mainly technical reasons: As a function of $\left(\left(x_{1}^{\prime }-x_{1}^{\prime \prime }\right),\left(x_{2}^{\prime }-x_{2}^{\prime \prime }\right)\right)$, *D*_CHEB_ fails to be differentiable everywhere, in contrast to *D*_EUCL_. Furthermore, when the amount of HWE disequilibrium is measured in terms of the Euclidean distance of (log *REH*, log *OR*) from the origin, only a single equivalence margin is involved in hypotheses formulation. Replacing Euclidean by Chebyshev distance leads to an equivalence region in the parameter space of (log *REH*, log *OR*) which is of rectangular rather than circular shape. This rectangle needs to be neither a square nor centered about the origin so that, in principle, 4 margins have to be specified numerically which considerably complicates the process of finding a consensus about how to make the testing problem fully precise. Insisting nevertheless on testing for equivalence in the sense that there holds $-\varepsilon_{1}^{\prime }<\text{log}\;\text{REH}<\varepsilon_{2}^{\prime }$ *and* $-\varepsilon_{1}^{\prime \prime }<\text{log}\;\text{OR}<\varepsilon_{2}^{\prime \prime }$ raises a problem for which an asymptotic solution is comparatively easy to derive exploiting the results of Section 3. The construction of such a test can be carried out through combining separate tests for equivalence in terms of log *REH* and log *OR* by means of the intersection-union principle. The details of this construction as well as an analysis of basic properties of the resulting procedure are left to a future publication.

From a technical perspective, the most innovative result of the paper is the derivation of an approximation to the rejection probability at the boundary of the range of the parameter of interest of a test based on a statistic shown to be asymptotically normal at any interior point of the parameter space. The corresponding sample size formula provides reasonable numerical accuracy and involves as the only non-elementary ingredient the inverse of a distribution function which can easily computed by means of standard tools of numerical analysis. For the implementation of the formula, a SAS/IML and a R script are available for download from the website hosting supporting materials (→ S1 Programs).

## Supporting information

S1 Appendix Proof of Proposition 1. Rigorous mathematical proof of the result stated as Proposition 1.
(PDF)

S2 Appendix Proof of Lemma 1. Derivation of the integral representation of the cumulative distribution function of an extended *χ*-distribution with 2 degrees of freedom.
(PDF)

S1 Programs Sample-size calculation by means of formula (28). Source-code listings for SAS and R users.
(TXT)

## References

[1] Panoutsopoulou K, Wheeler E. Key Concepts in Genetic Epidemiology. Methods Mol Biol. 2018;1793: 7–24. 10.1007/978-1-4939-7868-7_2 29876888

[2] Ziegler A, van Steen K, Wellek S. Investigating Hardy–Weinberg equilibrium in case–control or cohort studies or meta-analysis. Breast Cancer Res Treat. 2011; 128: 197–201. 10.1007/s10549-010-1295-z 21184275

[3] Ziegler A, König I. A statistical approach to genetic epidemiology: concepts and applications. Second edition Weinheim: Wiley-VCH; 2010.

[4] Ryckman K, Williams SM. Calculation and Use of the Hardy-Weinberg Model in Association Studies. Current Protocols in Human Genetics 2008; 1.18.1-1.18.11. 10.1002/0471142905.hg0118s57 18428419

[5] Wang J, Shete S. Testing Departure from Hardy-Weinberg Proportions In: Elston R. (eds) Statistical Human Genetics. Methods in Molecular Biology, vol 1666 Humana Press, New York, NY. 10.1007/978-1-4939-7274-6_6 28980243

[6] Loley C, Alver M, Assimes TL, Bjonnes A, Goel A, Gustafsson S, et al No association of coronary artery disease with X-chromosomal variants in vomprehensive international meta-analysis. Sci. Rep. 2016; 6: 35278 10.1038/srep35278 27731410 PMC5059659

[7] Graffelman J, Weir B. Testing for hardy–weinberg equilibrium at biallelic genetic markers on the x chromosome. Heredity. 2016; 116: 558–568. 10.1038/hdy.2016.20 27071844 PMC4868269

[8] Graffelman J. Exploring diallelic genetic markers: the Hardy-Weinberg package. J Stat Softw. 2015; 64(3): 1–22. 10.18637/jss.v064.i03

[9] Puig X, Ginebra J, Graffelman J. A Bayesian test for Hardy–Weinberg equilibrium of biallelic X-chromosomal markers. Heredity. 2017; 119: 226–236. 10.1038/hdy.2017.30 28900292 PMC5597779

[10] Wang P, Xu SQ, Wang BQ, Fung WK, Zhou JY. A robust and powerful test for case-control genetic association study on X chromosome. Stat Methods Med Res. 2018; 962280218799532 [Epub ahead of print]. 10.1177/0962280218799532 30232923

[11] You X-P, Zou Q-L, Li J-L, Zhou J-Y. Likelihood ratio test for excess homozygosity at marker loci on x chromosome. PLoS ONE. 2015; 10(14): 1–18. 10.1371/journal.pone.0145032 PMC4684405 26671781

[12] Graffelman J, Weir B. Multi-allelic exact tests for Hardy-Weinberg equilibrium that account for gender. Mol Ecol Resour. 2018;18(3): 461–473. 10.1111/1755-0998.12748 29288525 PMC5969302

[13] Zheng G, Joo J, Zhang C, Geller NL. Testing association for markers on the X chromosome. Genet Epidemiol. 2007;31(9): 834–43. 10.1002/gepi.20244 17549761

[14] Wellek S. Testing statistical hypotheses of equivalence and noninferiority. Second edition Boca Raton: Chapman & Hall/CRC; 2010.

[15] Wellek S. Tests for establishing compatibility of an observed genotype distribution with Hardy-Weinberg equilibrium in the case of biallelic locus. Biometrics. 2004; 60: 694–703. 10.1111/j.0006-341X.2004.00219.x 15339292

[16] Goddard KAB, Ziegler A, Wellek S. Adapting the logical basis of tests for Hardy-Weinberg equilibrium to the real needs of association tudies in human and medical genetics. Genet Epidemiol. 2009; 33: 569–580. 10.1002/gepi.20409 19235187

[17] Wellek S, Goddard KAB, Ziegler A. A confidence-limit-based approach to the assessment of hardy-weinberg equilibrium. Biom J. 2010; 52: 253–270. 10.1002/bimj.200900249 20394081

[18] Wellek S, Ziegler P. EQUIVNONINF: Testing for equivalence and noninferiority. R package version 1.0. 2017.

[19] Westlake WJ. Use of confidence intervals in analysis of comparative bioavailability trials. J Pharmacol Sci. 1972; 61: 1340–1341. 10.1002/jps.2600610845 5050398

[20] Berger RL. Multiparameter hypothesis testing and acceptance sampling. Technometrics. 1982; 24: 295–300. 10.2307/1267823

[21] Wellek S. Statistical methods for the analysis of two-arm non-inferiority trials with binary outcomes. Biom J. 2005; 47: 48–61. 10.1002/bimj.200410090 16395996

[22] Bishop Y, Fienberg S, Holland P. Discrete Multivariate Analysis. Cambridge, Mass: MIT Press; 1975.

[23] Farrington CP, Manning G. Test statistics and sample size formulae for comparative binomial trials with null hypothesis of non-zero risk difference or non-unit relative risk. Stat Med. 1990; 9: 1447–1454. 10.1002/sim.4780091208 2281232

[24] Billingsley P. Probability and measure. Third edition Hoboken: Wiley; 1995.

[25] NHGRI. GENEVA Genome-Wide association study of venous thrombosis. 2011. Available from: https://www.ncbi.nlm.nih.gov/projects/gap/cgi-bin/study.cgi?study_id=phs000289.v2.p1.

