# Supplementary material for: Testing for goodness rather than lack of fit of an X–chromosomal SNP to the Hardy-Weinberg model
Source: PLoS One. 2019 Feb 21;14(2):e0212344. doi: 10.1371/journal.pone.0212344 (PMC6383894; doi:10.1371/journal.pone.0212344)
Supplement: S1 Appendix — Rigorous mathematical proof of the result stated as Proposition 1. (PDF) [file pone.0212344.s001.pdf]

Supporting information to: Testing for goodness rather than lack of fit of a x-chromosomal SNP to the Hardy-Weinberg model

Stefan Wellek[1,2*], Andreas Ziegler[3,4,5]

1 Department of Biostatistics, CIMH Mannheim, Mannheim Medical School of the University of Heidelberg, D–68159 Mannheim, J5, Germany
2 Department of Medical Biostatistics, Epidemiology & Informatics, University Medical Center of the Johannes Gutenberg University Mainz, D–55101 Mainz, Germany
3 Institute of Medical Biometry and Statistics, University of Lübeck, Germany
4 StatSol, Moenring 2, 23560 Lübeck, Germany
5 School of Mathematics, Statistics and Computer Science, University of KwaZulu-Natal, Pietermaritzburg, South Africa

* stefan.wellek@zi-mannheim.de

A1: Proof of Proposition 1

By definition, the bivariate statistic whose joint asymptotic distribution we have to establish, is given by $(\hat{\Delta}^{\pm}_f, \hat{\Delta}^{\pm}_m) = \big(g_1(\hat{\pi}_1, \hat{\pi}_2, \hat{\pi}_3, \hat{p}_Y, 1-\hat{p}_Y), g_2(\hat{\pi}_1, \hat{\pi}_2, \hat{\pi}_3, \hat{p}_Y, 1-\hat{p}_Y\big)$, with

$$g_1(t_1, \ldots, t_5) = \log(t_2) - (\log(t_1) + \log(t_3))/2 - \log(2),$$
$$g_2(t_1, \ldots, t_5) = \log(t_1 + t_2/2) - \log(1 - t_1 - t_2/2)$$
$$- \log(t_4) + \log(t_5).$$

The mapping $(t_1, \ldots, t_5) \mapsto \mathbf{g}(t_1, \ldots, t_5) := \big(g_1(t_1, \ldots, t_5),\, g_2(t_1, \ldots, t_5)\big)$ from $\big\{(t_1, \ldots, t_5) \in (0,1)^5 \,\big|\, t_1 + t_2/2 < 1\big\}$ in $\mathbb{R}^2$ is differentiable everywhere in its domain of definition, and its matrix of partial derivatives of first order is easily computed to be

$$\frac{\partial \mathbf{g}}{\partial \mathbf{t}} = \begin{pmatrix} -\frac{1}{2t_1} & \frac{1}{t_1+t_2/2} + \frac{1}{1-t_1-t_2/2} \\ \frac{1}{t_2} & \frac{1}{2}\big(\frac{1}{t_1+t_2/2} + \frac{1}{1-t_1-t_2/2}\big) \\ -\frac{1}{2t_3} & 0 \\ 0 & -\frac{1}{t_4} \\ 0 & \frac{1}{t_5} \end{pmatrix} .$$

Weak convergence of $\sqrt{N}\big((\hat{\pi}_1, \hat{\pi}_2, \hat{\pi}_3, \hat{p}_Y, 1-\hat{p}_Y) - (\pi_1, \pi_2, \pi_3,\, p_Y, 1-p_Y)\big)$ to a normally distributed random vector with mean zero and covariance matrix (10) implies that $\sqrt{N}\big(\mathbf{g}(\hat{\pi}_1, \hat{\pi}_2,\, \hat{\pi}_3, \hat{p}_Y, 1-\hat{p}_Y) - \mathbf{g}(\pi_1, \pi_2, \pi_3, p_Y, 1-p_Y)\big) = \sqrt{N}\big((\hat{\Delta}^{\pm}_f, \hat{\Delta}^{\pm}_m) - (\Delta^{\pm}_f, \Delta^{\pm}_m)\big)$ converges in law to some (Z_1, Z_2), say, being Gaussian and centered about zero. Denoting the covariance matrix of (Z_1, Z_2) by $\mathbf{\Sigma_g}$, applying the multivariate delta-method (see, e.g., [1], § 14,6) yields:

$$\mathbf{\Sigma_g} = \left(\frac{\partial \mathbf{g}}{\partial \mathbf{t}}\bigg|_{\mathbf{t}=(\pi, p_Y, 1-p_Y)}\right)' \mathbf{\Sigma} \left(\frac{\partial \mathbf{g}}{\partial \mathbf{t}}\bigg|_{\mathbf{t}=(\pi, p_Y, 1-p_Y)}\right). \quad \text{(A.1)}$$

For the sake of keeping algebra as simple as possible, it is convenient to start evaluation of (A.1) with carrying out the second matrix multiplication first. After a few rearrangements of terms one can write

$$\mathbf{\Sigma}\left(\frac{\partial \mathbf{g}}{\partial \mathbf{t}}\Big|_{\mathbf{t}=(\pi,p_Y,1-p_Y)}\right)=\begin{pmatrix} -\frac{1}{2\lambda} & \frac{\pi_1}{\lambda(\pi_1+\pi_2/2)} \\ \frac{1}{\lambda} & \frac{\pi_2(2\pi_1+\pi_2-1)}{2\lambda(\pi_1+\pi_2/2)(\pi_1+\pi_2/2-1)} \\ -\frac{1}{2\lambda} & \frac{\pi_3}{\lambda(\pi_1+\pi_2/2-1)} \\ 0 & -\frac{1}{1-\lambda} \\ 0 & \frac{1}{1-\lambda} \end{pmatrix}. \tag{A.2}$$

Denoting the elements of $\mathbf{\Sigma_g}$ by $\sigma_{\mathbf{g}}^{j_1,j_2}$, $(j_1,j_2)\in\{1,2\}\times\{1,2\}$, we obtain using (A.2):

$$\sigma_{\mathbf{g}}^{1,1}=\Big(-\frac{1}{2\pi_1},\frac{1}{\pi_2},-\frac{1}{2\pi_3},0,0\Big)\Big(-\frac{1}{2\lambda},\frac{1}{\lambda},-\frac{1}{2\lambda},0,0\Big)'=\frac{1}{4\pi_1\lambda}+\frac{1}{\pi_2\lambda}+\frac{1}{4\pi_3\lambda}=\frac{1}{\lambda}\Big(\frac{\pi_1+\pi_3}{4\pi_1\pi_3}+\frac{1}{\pi_2}\Big),$$

$$\begin{aligned}\sigma_{\mathbf{g}}^{2,1}&=\Big(\frac{1}{\pi_1+\frac{\pi_2}{2}}+\frac{1}{1-\pi_1-\frac{\pi_2}{2}},\frac{1}{2(\pi_1+\frac{\pi_2}{2})}+\frac{1}{2(1-\pi_1-\frac{\pi_2}{2})},0,-\frac{1}{p_Y},\frac{1}{1-p_Y}\Big)\Big(\frac{-1}{2\lambda},\frac{1}{\lambda},\frac{-1}{2\lambda},0,0\Big)'\\&=-\frac{1}{2\lambda}\Big(\frac{1}{\pi_1+\frac{\pi_2}{2}}+\frac{1}{1-\pi_1-\frac{\pi_2}{2}}\Big)+\frac{1}{\lambda}\Big(\frac{1}{2(\pi_1+\frac{\pi_2}{2})}+\frac{1}{2(1-\pi_1-\frac{\pi_2}{2})}\Big)=0\,,\end{aligned}$$

$$\begin{aligned}\sigma_{\mathbf{g}}^{2,2}&=\Big(\frac{1}{\pi_1+\frac{\pi_2}{2}}+\frac{1}{1-\pi_1-\frac{\pi_2}{2}},\frac{1}{2(\pi_1+\frac{\pi_2}{2})}+\frac{1}{2(1-\pi_1-\frac{\pi_2}{2})},0,\frac{-1}{p_Y},\frac{1}{1-p_Y}\Big)\Big(\frac{\pi_1}{\lambda(\pi_1+\frac{\pi_2}{2})},\\&\qquad\frac{\pi_2(2\pi_1+\pi_2-1)}{2\lambda(\pi_1+\frac{\pi_2}{2})(\pi_1+\frac{\pi_2}{2}-1)},\frac{\pi_3}{\lambda(\pi_1+\frac{\pi_2}{2}-1)},\frac{-1}{1-\lambda},\frac{1}{1-\lambda}\Big)'\\&=\frac{\pi_1}{\lambda(\pi_1+\frac{\pi_2}{2})^2(1-\pi_1-\frac{\pi_2}{2})}-\frac{1}{4}\frac{\pi_2(2\pi_1+\pi_2-1)}{\lambda(\pi_1+\frac{\pi_2}{2})^2(1-\pi_1-\frac{\pi_2}{2})^2}+\frac{1}{(1-\lambda)p_Y(1-p_Y)}.\end{aligned}$$

$$\begin{aligned}&\Rightarrow\\&\Big[\sigma_{\mathbf{g}}^{2,2}-\frac{1}{(1-\lambda)p_Y(1-p_Y)}\Big]\\&\quad=\frac{4\pi_1(1-\pi_1-\frac{\pi_2}{2})+\pi_2(1-2\pi_1-\pi_2)}{4\lambda(\pi_1+\frac{\pi_2}{2})^2(1-\pi_1-\frac{\pi_2}{2})^2}\\&\quad=\frac{4(\pi_1+\frac{\pi_2}{2})(1-\pi_1-\frac{\pi_2}{2})-2\pi_2(1-\pi_1-\frac{\pi_2}{2})}{4\lambda(\pi_1+\frac{\pi_2}{2})^2(1-\pi_1-\frac{\pi_2}{2})^2}+\frac{\pi_2(1-2\pi_1-\pi_2)}{4\lambda(\pi_1+\frac{\pi_2}{2})^2(1-\pi_1-\frac{\pi_2}{2})^2}\\&\quad=\frac{4(\pi_1+\frac{\pi_2}{4})-4(1+\frac{\pi_2}{2})^2}{4\lambda(\pi_1+\frac{\pi_2}{2})^2(1-\pi_1-\frac{\pi_2}{2})^2}=\frac{1}{4}\frac{4\pi_1+\pi_2}{\lambda(\pi_1+\frac{\pi_2}{2})^2(1-\pi_1-\frac{\pi_2}{2})^2}-\frac{1}{\lambda(1-\pi_1-\frac{\pi_2}{2})^2}.\end{aligned}$$

Since, by definition, we have $\sigma_{\mathbf{g}}^{1,1}=\sigma_{f;\lambda}^2$, $\sigma_{\mathbf{g}}^{2,2}=\sigma_{m;\lambda}^2$, these equations complete the proof Proposition 1.

References

1. Bishop Y, Fienberg S, Holland P. Discrete Multivariate Analysis. Cambridge, Mass.: MIT Press; 1975.
