# Supplementary material for: Testing for goodness rather than lack of fit of an X–chromosomal SNP to the Hardy-Weinberg model
Source: PLoS One. 2019 Feb 21;14(2):e0212344. doi: 10.1371/journal.pone.0212344 (PMC6383894; doi:10.1371/journal.pone.0212344)
Supplement: S2 Appendix — Derivation of the integral representation of the cumulative distribution function of an extended χ-distribution with 2 degrees of freedom. (PDF) [file pone.0212344.s002.pdf]

Supporting information to: Testing for goodness rather than lack of fit of a x-chromosomal SNP to the Hardy-Weinberg model

Stefan Wellek[1,2*], Andreas Ziegler[3,4,5]

1 Department of Biostatistics, CIMH Mannheim, Mannheim Medical School of the University of Heidelberg, D–68159 Mannheim, J5, Germany
2 Department of Medical Biostatistics, Epidemiology & Informatics, University Medical Center of the Johannes Gutenberg University Mainz, D–55101 Mainz, Germany
3 Institute of Medical Biometry and Statistics, University of Lübeck, Germany
4 StatSol, Moenring 2, 23560 Lübeck, Germany
5 School of Mathematics, Statistics and Computer Science, University of KwaZulu-Natal, Pietermaritzburg, South Africa

* stefan.wellek@zi-mannheim.de

A2: Proof of Lemma 1

Let (Z_1, Z_2) be independent with respect to some fixed probability measure $P(\cdot)$, with marginal distributions $\mathcal{N}(0,1)$ and $\mathcal{N}(0,c^2)$. Then, for any $q > 0$, one can write:

$$P\big[\sqrt{Z_1^2 + Z_2^2} \leq q\big] = P\big[Z_2^2 \leq q^2 - Z_1^2\big]$$

$$= \int_{-q}^{q} P\big[Z_2^2 \leq q^2 - Z_1^2 \big| Z_1 = z_1\big] dP_{Z_1}(z_1)$$

$$= \int_{-q}^{q} P_{Z_2}\big[-\sqrt{q^2 - z_1^2} \leq Z_2 \leq \sqrt{q^2 - z_1^2}\big] dP_{Z_1}(z_1)$$

$$= \int_{-q}^{q} \Big(2\Phi\big[\sqrt{q^2 - z_1^2}/c\big] - 1\Big) d\Phi(z_1) = 2\int_{-q}^{q} \Phi\Big(\frac{1}{c}\sqrt{q^2 - z_1^2}\Big)\phi(z_1)\, dz_1 - [2\Phi(q) - 1]\,.$$

According to the definition of $\mathcal{Q}_c(\cdot)$, we have $\mathcal{Q}_c(q) = P\big[\sqrt{Z_1^2 + Z_2^2} \leq q\big]$, so that the proof of Lemma 1 is complete.
